# Endogenous Ethylene Concentration Is Not a Major Determinant of Fruit Abscission in Heat-Stressed Cotton (*Gossypium hirsutum* L.)

**DOI:** 10.3389/fpls.2017.01615

**Published:** 2017-09-21

**Authors:** Ullah Najeeb, Muhammad Sarwar, Brian J. Atwell, Michael P. Bange, Daniel K. Y. Tan

**Affiliations:** ^1^Faculty of Science, Plant Breeding Institute, Sydney Institute of Agriculture, School of Life and Environmental Sciences, University of Sydney Sydney, NSW, Australia; ^2^Agronomic Research Institute, Ayub Agricultural Research Institute Faisalabad, Pakistan; ^3^Department of Biological Sciences, Faculty of Science and Engineering, Macquarie University Sydney NSW, Australia; ^4^Commonwealth Scientific and Industrial Research Organisation Agriculture and Food, Australian Cotton Research Institute Narrabri, NSW, Australia

**Keywords:** elevated temperature, ethylene manipulation, heat shock, photosynthesis, pollen germination

## Abstract

We investigated the role of ethylene in the response of cotton to high temperature using cotton genotypes with genetically interrupted ethylene metabolism. In the first experiment, Sicot 71BRF and 5B (a lintless variant with compromised ethylene metabolism) were exposed to 45°C, either by instantaneous heat shock or by ramping temperatures by 3°C daily for 1 week. One day prior to the start of heat treatment, half the plants were sprayed with 0.8 mM of the ethylene synthesis inhibitor, aminoethoxyvinylglycine (AVG). In a subsequent experiment, Sicot 71BRF and a putatively heat-tolerant line, CIM 448, were exposed to 36 or 45°C for 1 week, and half the plants were sprayed with 20 μM of the ethylene precursor, 1-aminocyclopropane-1-carboxylic acid, (ACC). High temperature exposure of plants in both experiments was performed at the peak reproductive phase (65–68 days after sowing). Elevated temperature (heat shock or ramping to 45°C) significantly reduced production and retention of fruits in all cotton lines used in this study. At the termination of heat treatment, cotton plants exposed to 45°C had at least 50% fewer fruits than plants under optimum temperature in all three genotypes, while plants at 36°C remained unaffected. Heat-stressed plants continued producing new squares (fruiting buds) after termination of heat stress but these squares did not turn into cotton bolls due to pollen infertility. *In vitro* inhibition of pollen germination by high temperatures supported this observation. Leaf photosynthesis (*P*_n_) of heat-stressed plants (45°C) measured at the end of heat treatments remained significantly inhibited, despite an increased leaf stomatal conductance (*g*_s_), suggesting that high temperature impairs *P*_n_ independently of stomatal behavior. Metabolic injury was supported by high relative cellular injury and low photosystem II yield of the heat-stressed plants, indicating that high temperature impaired photosynthetic electron transport. Both heat shock and ramping of heat significantly reduced ethylene release from cotton leaf tissues measured at the end of heat treatment but modulating ethylene production via AVG or ACC application had no significant effect on fruit production or retention in heat-stressed cotton plants. Instead, high temperature accelerated fruit abortion by impairing pollen development and/or restricting leaf photosynthesis.

## Introduction

With rising atmospheric temperatures, there has been an increased concern about protecting crops from extreme weather events such as, heat shock (short bursts of very high temperatures), which are expected to become more common in near future (Zheng et al., 2012). For example, Australia has been experiencing an increased frequency of very hot (>40°C) daytime temperatures since the 1990s (CSIRO and Bureau of Meteorology, 2012). Cotton is widely cultivated under hot, semi-arid climatic conditions hence crops often experience yield losses due to episodes of high air temperatures during reproductive phase. Optimum temperature for cotton growth has been determined as 20–30°C (Reddy et al., 1991a), with temperatures above this optimum negatively influencing productivity. For example, a lint yield reduction of 110 kg ha^−1^ is expected for each 1°C rise in maximum day temperature (Singh et al., 2007) due to accelerated fruit shedding during the post-stress period (Ehlig and Lemert, 1973).

Elevated temperature influences various processes during reproductive growth such as, pollen formation, pollen germination, and fertilization, with failure leading to abscission of reproductive structures (Kakani et al., 2005). For example, day temperatures above 40°C can induce abscission of cotton flowers and young squares developed during heat stress (Reddy et al., 1991a). In addition to pollen abortion, high temperature induces lint yield losses by influencing key physiological processes such as, photosynthesis, stomatal behavior and cell membrane thermo-stability (Bibi et al., 2008; Cottee et al., 2010). In brief, yield losses in heat-stressed cotton crops could be result of impaired development of reproductive parts (pollen or pistil) and/or competition for photo-assimilates (Zhao et al., 2005).

Fruit abscission is mediated by enzymes that hydrolyse pectin-rich middle lamella in the abscission zone (Sawicki et al., 2015); ethylene is invoked as a key factor that triggers the expression of these hydrolytic genes (Zhu et al., 2011). Furthermore, ethylene release is accelerated by stress factors such as, temperature, excessive light, and waterlogging (Jackson, 1985; Hyodo, 1991). Thus, we hypothesized that ethylene plays a role in fruit abscission in cotton stressed at high temperatures. Previously, we observed a strong positive correlation between waterlogging-induced fruit abscission and ethylene concentrations in cotton tissues (Najeeb et al., 2015). Such stress-induced damage can be modified by regulation of the synthesis (Grichko and Glick, 2001) or perception (Wilkinson et al., 1997) of ethylene through genetic manipulation. Furthermore, ethylene metabolism can be suppressed by blockers of ethylene synthesis or perception such as, aminoethoxyvinylglycine (AVG) and 1-methylcyclopropene (1-MCP), effectively increasing tolerance to abiotic stresses in cotton (Bange et al., 2010; Kawakami et al., 2013). Earlier studies suggested positive effects of modulating ethylene production through 1-MCP on different crops including soybean and wheat (Hays et al., 2007; Djanaguiraman et al., 2011). Variable effects 1-MCP application have been observed on cotton crops e.g., Kawakami et al. (2013) observed increased lint yield in cotton in response to 1-MCP application but Scheiner et al. (2007) suggested no effect of 1-MCP on cotton lint yield.

The present study explores the relationship between ethylene metabolism and fruit abscission in heat-stressed cotton, testing the hypothesis that elevated ethylene levels are involved in heat-induced fruit loss. The first experiment attempted to minimize heat-induced fruit abscission by manipulating ethylene production using: (1) AVG to block ethylene synthesis and (2) the lintless cotton mutant (5B), which produces very few fibers in the boll. Since fiber development and elongation in cotton is regulated by ethylene (Shi et al., 2006), it is proposed that ethylene metabolism is impaired in this cotton mutant. Earlier studies suggested a lesion in the pathway of ethylene synthesis in developing ovules of lintless mutants (Wang et al., 2011; Gilbert et al., 2013). While we cannot confirm the location of the genetic block in mutant 5B, it has a definitive phenotype and thus constituted an ideal genetic tool to test the role of ethylene under heat. In a separate glasshouse experiment, the effects of an ethylene precursor 1-aminocyclopropane-1-carboxylic acid (ACC) were investigated in two cotton cultivars with naturally contrasting heat tolerance to see whether induced ethylene release exacerbated sensitivity to heat. The main objectives of these experiments were (1) to study how cotton plants respond to elevated temperatures (heat shock and ramping heat) and (2) to explore the impact of ethylene on heat tolerance in cotton.

## Materials and methods

### Plant material and growth conditions

#### Experiment 1

Two cotton genotypes, Sicot 71 BRF and 5B line, were used in this glasshouse experiment. Sicot 71BRF is a commercial cotton cultivar [*Gossypium hirsutum* L. (Bollgard II® Roundup Ready Flex®), CSIRO Australia; (Stiller, 2008)] that is widely cultivated in Australian cotton growing regions. The 5B line was originally separated from a fully linted cotton cultivar B1278 as a spontaneous mutant, having little or no lint on the seeds (Dr. Alistair Low unpublished, CSIRO Irrigation Research, Griffith, NSW). Consistent with a disruption to ethylene metabolism, the 5B mutant line exhibited a tendency to retain fruits in response to soil waterlogging (Najeeb et al., unpublished data), while fruit abscission is normally strongly associated with increased ethylene production from vegetative tissues of waterlogged cotton plants (Najeeb et al., 2015).

Seeds were sown in plastic pots (25 × 20 cm; height × diameter) containing finely mixed red silt loam Ferrosol soil from Robertson, NSW, Australia. Plants were grown under similar climatic and cultural conditions as reported in an earlier study (Najeeb et al., 2015). In brief, plants were grown at 28/20°C day/night temperature, 50–70% relative humidity, 14/10 light/dark photoperiod under natural light. The light intensity during the day cycle was maintained to a minimum of 400 μmol m^−2^ s^−1^ using supplemented light (Philips Contempa High Pressure Sodium lamps). At peak reproductive growth phase (65 days after sowing, DAS), the plants were either transferred to growth cabinets at 45/30°C, day/night temperature directly (heat shock) or to growth cabinets where day temperature was increased to 45°C by an incremental rise of 3°C per day (e.g., 33, 36, 39, 42, and 45°C-ramping heat). Plants were transferred to the high temperature growth cabinets at 9:00–9:30 a.m., and were exposed to 45°C day temperature for 1 week. Notably, plants under ramping heat experienced a cumulative 11 days of temperatures higher than optimum (cf. 7 days for the heat-shock treatment). Control plants were grown under optimal temperature (28/20°C, day/night). Soil was maintained at field capacity while humidity levels in growth chambers were kept at >50% by placing water-filled plastic trays on the cabinet floor.

One day prior to the start of heat treatments, the plants in each set were sprayed with either 0.8 or 0 mM (water) AVG formulated as ReTain® (Sumitomo Chemicals Australia) + one drop of detergent (Supplementary Table 1). AVG application rates have been optimized in earlier glasshouse and field experiments (Bange et al., 2010; Najeeb et al., 2016) and the rate applied here was effective in suppressing ethylene production and fruit loss in waterlogged cotton crops (Najeeb et al., 2015).

#### Experiment 2

In this experiment, a relatively heat-tolerant cotton cultivar CIM 448 (Rahman, 2005) was used along with Sicot 71BRF. The plants were exposed to either 45/30°C or 36/30°C day/night temperature (each applied as a heat shock without ramping) for 7 days at the peak reproductive phase (68 DAS). Control plants were grown under 28/20°C day/night temperature. As the plants were exposed to heat shock, an immediate effect of heat on ethylene synthesis was expected. To modulate ethylene synthesis, 20 μM ACC was sprayed on cotton plants (Supplementary Table 1). Plants readily convert ACC into ethylene so ACC was applied on 2 consecutive days, namely 10 μM ACC 1 day prior to heat treatment and 10 μM at the start of heat treatment. After heat-treatment, all plants were returned to optimum conditions (28/20°C day/night temperature) to study the recovery in reproductive physiology.

### Fruit production and retention

Data on fruit production and retention were collected the day before (−1 day) then 8 and 15 days after heat treatments (DHT). With no significant variation in the number fruits at −1 and 8 DHT, data are presented only for −1 and 15 DHT. All plants were mapped for fruit number and type (squares and green bolls) on individual fruiting position and branches. Pollinated flowers and bolls were assigned as green bolls and young non-pollinated flowers as squares. Fruit retention (FR) was calculated from the ratio of retained fruits to total fruiting sites on each plant.

### Leaf gas exchange and chlorophyll fluorescence

Leaf CO_2_ exchange parameters such as, photosynthesis (*P*_n_) and stomatal conductance (*g*_s_) were measured at the termination of heat treatments. Data were collected from the youngest fully expanded leaves between 9:00 and 13:00 h using a Li-6400 portable photosynthesis system (Li-Cor Ltd, Lincoln, NE, USA) with a pulse-amplitude modulated (PAM) leaf chamber head. The gas exchange measurements were recorded at 1,800 μmol photon m^−2^ s^−1^ photosynthetically active radiation (PAR) and a CO_2_ concentration of 400 μmol mol^−1^. Temperature inside the leaf chamber was adjusted according to the corresponding treatment temperature e.g., 28, 36, or 45°C.

Chlorophyll fluorescence was measured using a photosynthesis yield analyser (MINI- PAM, Walz, Effeltrich, Germany).

### Relative cell membrane injury

Leaf discs (14-mm diameter) were excised from the interveinal section of fully expanded leaves between 13:00 and 14:30 h at the end of heat treatments. The discs were triple rinsed with distilled water to remove any exogenous electrolytes, and then placed into glass vials containing 2 mL of MilliQ water. Vials were incubated in water bath at 25 and 55°C. Electrical conductivity (EC) was measured using a waterproof ECT calibrated conductivity meter before incubation (IEC) and after incubation (FEC).

Relative cellular injury (*RCI*%) was calculated according to Cottee et al. (2007) using the following equation:

RCI= 1− [1− (IECt/FECt)/1− (IECc/FECc) ×100

Where IECt and FECt are the initial and final EC values of heat-treated vials, respectively, while IECc and FECc are the initial and final EC values of control vials, respectively.

### *In Vitro* pollen germination

Flowers used for pollen viability assay were collected from the first fruiting position during peak reproductive growth phases (65–75 DAS) on a daily basis during both experiments. Pollen was collected between 8:00 and 9:00 a.m. and directly sprinkled on solidified nutrient media (Taylor, 1972), consisting of 1.5 g agar, 30 g sucrose, and (mg) KNO_3_ (5.3), MnSO_4_ (51.7), H_3_BO_3_ (10.3), MgSO_4_·7H_2_O (10.3) in 100 mL of deionised water (Kakani et al., 2005). Each replicate comprised three flowers from the same plant, with four plants per treatment for each genotype. Pollen germination percentage was calculated by counting the proportion of pollen grains and pollen tube length was measured using ImageJ software.

### Ethylene measurements

Youngest fully expanded leaves were collected from heated and control plants (three leaves per plant) 24, 48 h, and 7 d after heat treatment. In addition, ethylene was measured from leaf tissues 12 h after spraying ACC. Significantly higher ethylene concentrations were recorded from ACC-treated plants after 12 and 24 h of treatment under optimum temperatures but no change in ethylene concentration was observed in the later measurements. With no significant variation in the ethylene levels at different measurement times (except ACC-treated plants at 12 and 24 h under optimum temperatures), data were only presented for plants 7 d after heat treatment. This ethylene measurement time was selected from our earlier observations, where high temperature caused maximum damage to cotton plants. Ethylene concentrations from leaf tissues were measured using a protocol adopted by Najeeb et al. (2015). Leaf tissues were placed in 50-mL glass vials containing rubber septa. One milliliter gas samples were withdrawn using gas-tight syringes after 20–30 min (Jackson and Campbell, 1976) and injected into PYE series 104 gas chromatograph fitted with a flame ionization detector (FID) and equipped with activated aluminum coated glass column. Fresh biomass of the leaf tissues was determined after ethylene detection, and ethylene synthesis rates were calculated as nmol g^−1^ FW h^−1^.

### Data analysis

Data for different growth parameters was statistically analyzed by JMP v. 9 (SAS Institute, Cary, NC, USA) statistical program. A linear mixed model REML (Residual Maximum Likelihood) was applied to assess the individual and interactive effects of temperature, genotype, AVG, and ACC, while the respective means were compared using Tukey's HSD (honestly significant difference) test. Data for each experiment and measurement time were separately analyzed.

To identify the parameters that best describe high temperature-induced fruit loss in cotton, a principal component analysis (PCA) was performed. Values of *P*_n_, leaf ethylene concentrations, RCI, and number of green bolls of the three cotton cultivars under various treatment conditions were included in the PCA. This analysis estimates and then ranks principal components (PC) for contribution to the variation in data by consolidating the relationships among measured physiological variables.

## Results

### Fruit production

**Figure 1:**
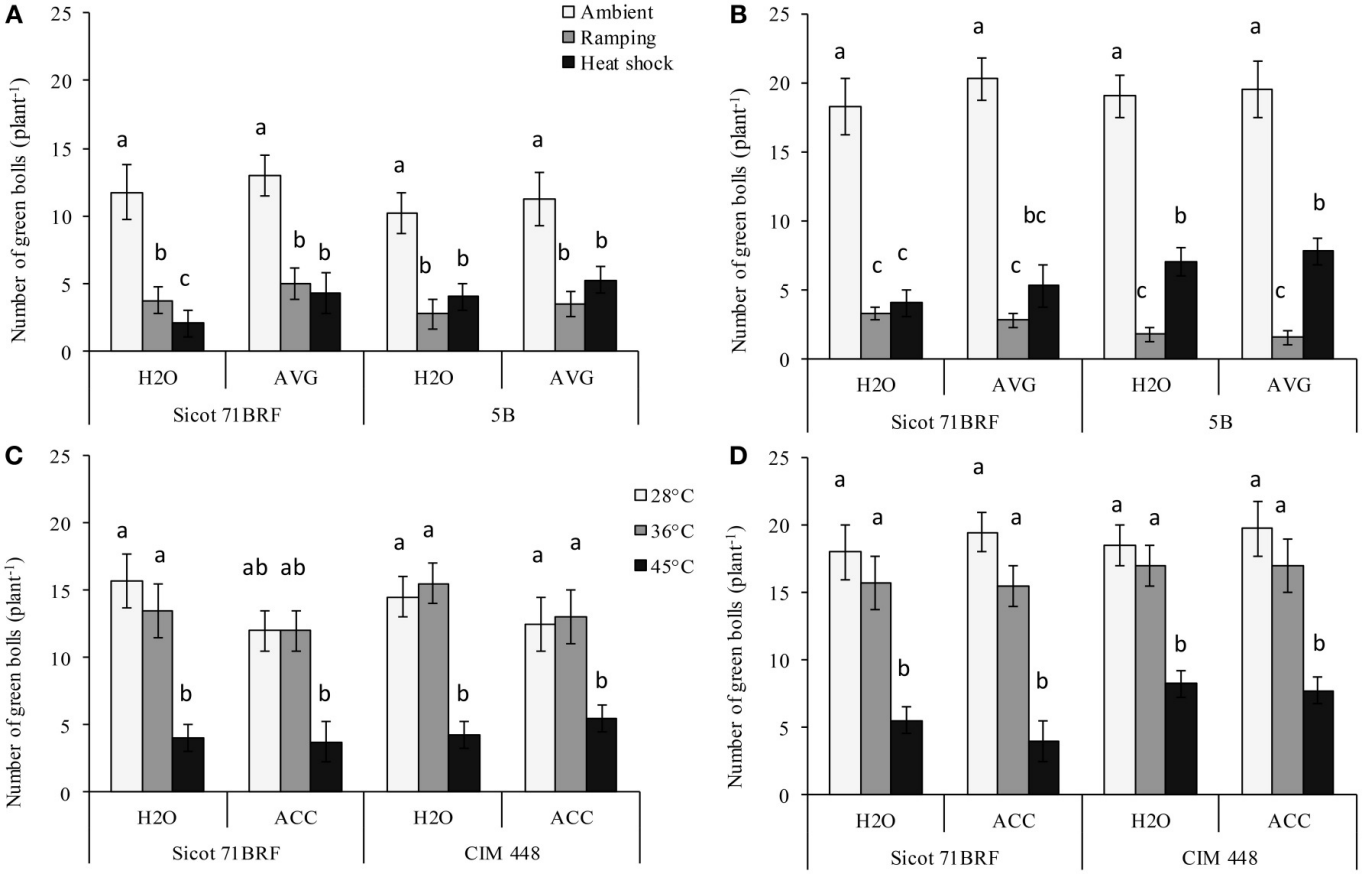
Effect of aminoethoxyvinylglycine (AVG) on number of green bolls (pollinated flowers + bolls) in cotton genotypes Sicot 71BRF and 5B exposed to heat shock (45°C) and ramping heat (gradual rise in temperature to 45°C) for 7 days. Data were collected **(A)** on the last day of heat treatment and **(B)** 15 days of recovery after the heat treatment. Values are the mean of four independent replications, with the error bars representing SE. Effect of 1-aminocyclopropane-1-carboxylic acid (ACC) on the number of green bolls in cotton genotypes Sicot 71BRF and CIM 448 exposed to heat shock (45 and 36°C) for 7 days. Data were collected **(C)** on the last day of heat treatment and **(D)** 15 days of recovery after the heat treatment. Values are the mean of four independent replications with the error bars representing SE and sample size = 4. Means sharing same letters within each figure are not significantly different at α = 0.05.

The numbers of green bolls (pollinated flowers + bolls) were significantly reduced with exposure to heat shock (45°C) or ramping high temperature (Supplementary Table 2). At the end of heat treatment, Sicot 71BRF plants exposed to heat (shock or ramping heat) had 80% fewer green bolls (averaged across AVG and H_2_O treatments) compared with plants under optimum temperature (Figure 1A). Similarly, 5B plants showed a 52–66% reduction in green bolls in response to heat shock and ramping heat, respectively, compared with the plants under control temperature (Figure 1A). In the 15 d after heat-shock, plants produced new green bolls, albeit fewer than the controls. On the other hand, after a period of ramping heat up to 45°C, plants did not produce any additional green bolls over the subsequent 15 d (Figure 1B). AVG had no significant effect on numbers of green bolls in 5B or Sicot 71BRF under any treatment condition (Figure 1A).

In the 2nd experiment, Sicot 71BRF displayed a similar response to 45°C heat shock as observed in Experiment 1 i.e., stressed plants experienced 75 and 69% reduction (averaged across ACC and H_2_O treatments) in green bolls at −1 and 15 DHT, respectively (Figures 1C,D). In addition, plants exposed to 36°C produced significantly fewer green bolls than plants at optimal temperature at 15 DHT, although the reduction was substantially less than that was seen at 45°C (Figures 1C,D). In CIM 448, a significant reduction in green bolls was also recorded in response to 45°C both at 1 and 15 DHT. In contrast, CIM 448 plants exposed to 36°C showed no significant reduction in green bolls at −1 or 15 DHT (Figures 1C,D). ACC significantly reduced green bolls of Sicot 71 BRF at −1 DHT under all temperatures but it induced more green bolls in both cultivars at 15 DHT under optimum temperature (Figures 1C,D). This increased green boll production was observed mainly at the top of the plant canopy.

**Figure 2:**
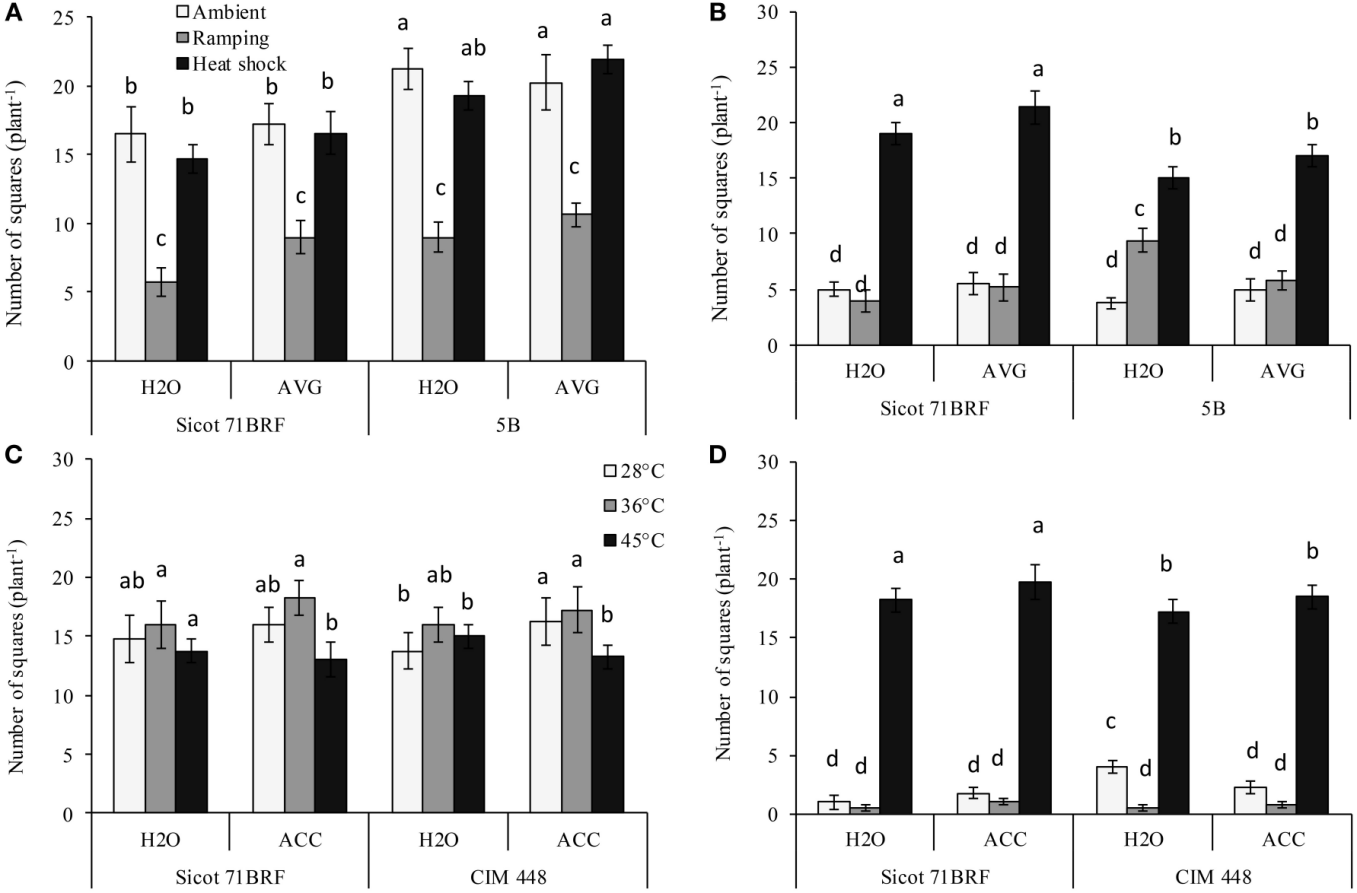
Effect of aminoethoxyvinylglycine (AVG) on the number of squares (non-pollinated flowers) in cotton genotypes Sicot 71BRF and 5B exposed to heat shock (45°C) and ramping heat (gradual rise in temperature to 45°C) for 7 days. Data were collected **(A)** on the last day of heat treatment and **(B)** 15 days of recovery after the heat treatment. Values are the mean of four independent replications, with the error bars representing SE and sample size = 4. Effect of 1-aminocyclopropane-1-carboxylic acid (ACC) on the number of squares in cotton genotypes Sicot 71BRF and CIM 448 exposed to heat shock (45 and 36°C) for 7 days. Data were collected **(C)** on the last day of heat treatment and **(D)** 15 days of recovery after the heat treatment. Values are the mean of four independent replications, with the error bars representing SE and sample size = 4. Means sharing same letters within each figure are not significantly different at α = 0.05.

The number of squares in both genotypes (Sicot 71BRF and 5B) was significantly reduced by heat shock or ramping heat at −1 DHT. Significantly greater loss of squares resulted from ramping heat (62% loss, averaged across both genotypes) than heat shock (15% loss, averaged across both genotypes; Figure 2A). In Sicot 71BRF, the number of squares in control and ramping-heat plants were significantly reduced at 15 DHT, although, heat-shock plants produced significantly more squares than both control and ramping heat plants over this period. In contrast, all heat-stressed 5B plants (heat shock and ramping heat without AVG spraying) produced significantly more squares than the control at 15 DHT (Figure 2B). AVG-treated heat-stressed (heat shock or ramping heat) Sicot 71BRF plants bore significantly more squares than their respective non-AVG treated plants at −1 DHT but the effect at 15 DHT was significant only on heat-shock plants. AVG had no significant effect on number of squares in 5B plants.

By the end of heat treatment (−1 DHT) in the 2nd experiment, heat shock at 45°C reduced number of squares in both genotypes (Sicot 71BRF and CIM 448) while plants exposed to 36°C produced marginally more squares than control plants (Figure 2C). In stark contrast, 45°C-treated plants produced significantly more squares than plants at 36 and 28°C 15 DHT (Figure 2D). ACC-treated plants of both cotton genotypes exposed to 28 and 36°C treatments produced slightly more squares at −1 DHT than non-ACC treated plants (*P* = 0.047) but the effect was non-significant at 15 DHT, as the plants supported only a few squares at this stage (Figures 2C,D).

### Fruit retention

**Figure 3:**
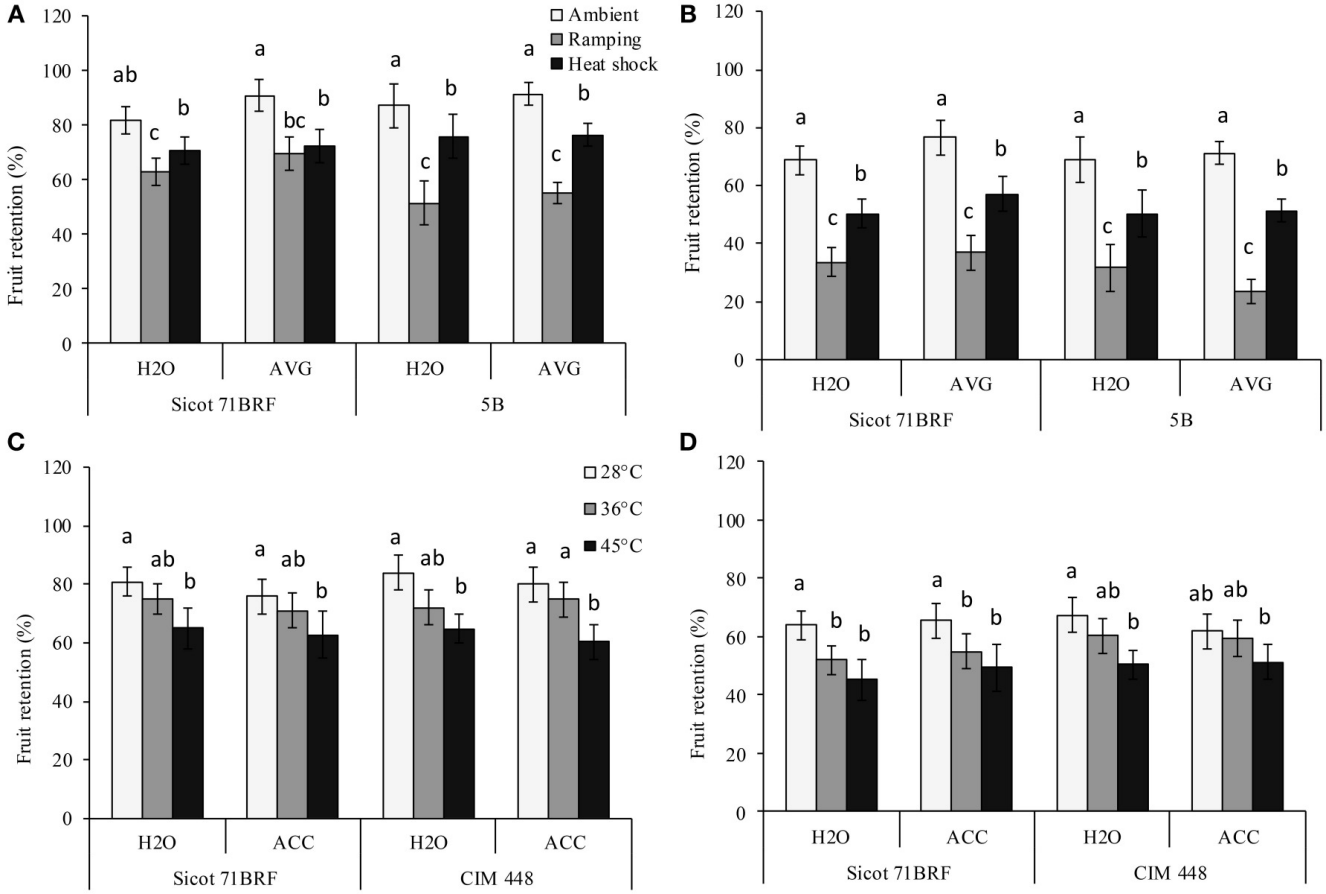
Effect of aminoethoxyvinylglycine (AVG) on fruit retention in cotton genotypes Sicot 71BRF and 5B exposed to heat shock (45°C) and ramping heat (gradual rise in temperature to 45°C) for 7 days. Data were collected **(A)** on the last day of heat treatment and **(B)** 15 days of recovery after the heat treatment. Values are the mean of four independent replications, with the error bars representing SE and sample size = 4. Effect of 1-aminocyclopropane-1-carboxylic acid (ACC) on fruit retention in cotton genotypes Sicot 71BRF and CIM 448 exposed to heat shock (45 and 36°C) for 7 days. Data were collected **(C)** on the last day of heat treatment and **(D)** 15 days of recovery after the heat treatment. Values are the mean of four independent replications, with the error bars representing SE and sample size = 4. Means sharing same letters within each figure are not significantly different at α = 0.05.

High temperature (heat shock or ramping) significantly reduced fruit retention (FR) in all the studied genotypes. The greatest reduction in FR was observed under ramping heat, which caused 29 and 42% reduction in FR of Sicot 71BRF and 5B, respectively, compared with their respective controls (optimum temperature) at −1 DHT. Similarly, plants exposed to heat shock (45°C) had 20% (averaged across all the three studied genotypes) lower FR compared with their respective control at −1 DHT (Figures 3A,C). Further reduction in FR of heat-stressed plants of all the studied genotypes was observed at 15 DHT (Figures 3B,D). Although, exposure to 36°C caused no significant effect on FR of Sicot 71BRF and CIM 448 at −1 DHT, it significantly reduced FR of Sicot 71BRF at 15 DHT. AVG slightly increased (Genotype × Temp × AVG; *P* < 0.05) FR of Sicot 71BRF plants under ambient conditions only but no significant effect of ACC was observed on FR of any genotype under any heat treatment (Supplementary Table 3).

### Leaf gas exchange and chlorophyll fluorescence

Elevated temperature significantly reduced photosynthetic capacity of both cotton genotypes (Sicot 71BRF and 5B), although the response to heat stress varied in these two genotypes (Genotype × Temp, *P* < 0.05; Supplementary Table 4). Heat shock caused relatively greater reduction in *P*_n_ of Sicot 71 BRF (20% lower than control) than in 5B (13% lower than control; Table 1). By contrast, 5B plants exposed to ramping heat experienced a greater reduction in *P*_n_ (49% lower than control) than Sicot 71 BRF (18% lower than control; Table 1). Along the same lines, 36°C had no significant effect on *P*_n_ of Sicot 71BRF leaves, but it significantly increased *P*_n_ in CIM 448 leaves. ACC had no significant effect on *P*_n_ of cotton genotypes under any treatment condition (Table 2).

**Table 1:** Changes in various physiological components of different cotton genotypes in response to heat shock (45°C), ramping heat (45°C), and aminoethoxyvinylglycine (AVG) application in Experiment 1.

**Genotype**	**Treatment**	***P*_n_ (μmol CO_2_ m^−2^ s^−1^)**	***g*_s_ (mol H_2_O m^−2^ s^−1^)**	**PSII yield**	**RCI (%)**
Sicot 71BRF	Control+AVG	29.4 (2.5)a	0.48 (0.05)c	0.42 (0.04)a	77.0 (6.1)b
	Control+H_2_O	28.3 (2.2)a	0.51 (0.05)c	0.42 (0.05)a	76.9 (6.8)b
	Heat shock+AVG	24.2 (1.8)ab	0.63 (0.07)b	0.29 (0.03)bc	93.5 (7.3)a
	Heat shock+H_2_O	23.1 (2.0)b	0.73 (0.07)ab	0.32 (0.03)b	89.2 (7.2)a
	Ramping+AVG	20.0 (2.1)b	0.84 (0.07)a	0.31 0.03)b	91.3 (7.7)a
	Ramping+H_2_O	19.4 (1.7)b	0.71 (0.06)ab	0.25 (0.03)c	94.7 (6.5)a
5B (Lintless mutant)	Control+AVG	26.6 (1.9)a	0.37 (0.04)d	0.46 (0.05)a	75.4 (6.4)b
	Control+H_2_O	27.3 (2.2)a	0.42 (0.05)cd	0.43 (0.05)a	82.1 (7.1)ab
	Heat shock+AVG	23.6 (1.7)b	0.65 (0.05)b	0.36 (0.04)b	92.8 (7.3)a
	Heat shock+H_2_O	21.9 (2.1)b	0.70 (0.06)b	0.33 (0.03)b	96.0 (6.6)a
	Ramping+AVG	11.2 (1.2)c	0.20 (0.05)e	0.21 (0.03)cd	94.2 (6.9)a
	Ramping+H_2_O	12.8 (1.2)c	0.19 (0.05)e	0.18 (0.02)d	97.3 (8.3)a

*Data were collected from the topmost fully expanded leaves at the termination of 7 d heat treatment. g_s_, stomatal conductance (mol H_2_O m^-2^ s^-1^), P_n_, rate of photosynthesis (μmol CO_2_ m^-2^ s^-1^), PSII yield, photosystem II yield, RCI, % relative cell injury. Values are the mean of four independent replications with standard error in parenthesis (brackets) and sample size = 4. Means sharing same letters within each column are not significantly different at α = 0.05*.

**Table 2:** Changes in various physiological componets of different cotton genotypes in response to heat shock (36 and 45°C) and 1-aminocylopropane-1-carboxlyic acid (ACC) application in experiment 2.

	**Treatment**	***P*_n_ (μmol CO_2_ m^−2^ s^−1^)**	***g*_s_ (mol H_2_O m^−2^ s^−1^)**	**PSII yield**	**RCI (%)**
Sicot 71BRF	Control+ACC	27.5 (2.1)a	0.47 (0.0)5c	0.46 (0.05)a	79.3 (7.0)b
	Control+H_2_O	28.8 (2.1)a	0.44 (0.05)c	0.47 (0.05)a	77.7 (6.3)b
	36°C+ACC	30.4 (2.5)a	0.68 (0.05)b	0.44 (0.05)a	89.0 (7.5)a
	36°C+H_2_O	29.1 (2.4)a	0.58 (0.05)b	0.45 (0.05)a	86.6 (7.3)ab
	45°C+ACC	20.9 (2.5)b	0.94 (0.08)a	0.33 (0.04)b	93.2 (7.7)a
	45°C+H_2_O	20.5 (2.5)b	0.87 (0.07)a	0.31 (0.04)b	91.0 (7.6)a
CIM 448	Control+ACC	26.7 (2.0)a	0.48 (0.05)c	0.47 (0.05)a	75.1 (6.2)b
	Control+H_2_O	24.8 (2.2)a	0.45 (0.04)c	0.48 (0.05)a	73.3 (6.1)b
	36°C+ACC	31.3 (2.6)a	0.72 (0.05)b	0.42 (0.05)a	86.1 (7.3)ab
	36°C+H_2_O	29.2 (2.3)a	0.64 (0.05)b	0.44 (0.05)a	80.8 (7.0)ab
	45°C+ACC	18.4 (2.1)b	0.88 (0.06)a	0.34 (0.04)b	91.6 (7.6)a
	45°C+H_2_O	19.9 (2.1)b	0.96 (0.07)a	0.35 (0.04)b	93.5 (7.5)a

*Data were collected from the topmost fully expanded leaves at the termination of 7 d heat treatment. g_s_, stomatal conductance (mol H_2_O m^-2^ s^-1^), P_n_, rate of photosynthesis (μmol CO_2_ m^-2^ s^-1^), PSII yield, photosystem II yield, RCI, % relative cell injury. Values are the mean of four independent replications with standard error in parenthesis (brackets) and sample size = 4. Means sharing same letters within the column are not significantly different at α = 0.05*.

As for *P*_n_, stomatal conductance (*g*_s_) of cotton genotypes was variably affected by heat shock and ramping heat (Supplementary Table 4). Sicot 71BRF plants had 25 and 22% higher *g*_s_ under heat shock and ramping heat stress, respectively, compared with control plants (Table 1) while *g*_s_ of 5B plants significantly increased (49% higher than control) in response to heat-shock and but decreased (68% lower than control) under ramping heat (Table 1). Sicot 71BRF plants exposed to 45 and 36°C had 32 and 98% higher *g*_s_, respectively, than control. In a similar manner to Sicot 71BRF, CIM 448 plants exhibited 48 and 92% increase in *g*_s_ under 45 and 36°C, respectively, compared with control (Table 2). AVG and ACC application had no significant effect on *g*_s_ of cotton genotypes under optimum conditions but AVG slightly increased *g*_s_ of Sicot 71BRF under ramping heat and decreased under heat shock (Supplementary Table 4).

Heat shock (45°C) equally inhibited the PSII yield of cotton leaves in both glasshouse experiments, causing 25% (averaged across three genotypes) reduction in PSII yield compared with their respective controls (Tables 1, 2). Ramping heat also significantly reduced PSII yield of cotton leaves and the reduction was relatively greater in 5B (58% reduction over control) than Sicot 71BRF (40% reduction over control). In contrast, no significant effect of 36°C was observed on PSII yield of Sicot 71BRF or CIM 448 leaves. ACC or AVG application had no significant effect on PSII yield of cotton leaves under any treatment conditions (Supplementary Table 4).

### Relative cell membrane injury

High temperature treatment (heat shock and ramping heat) significantly increased *RCI* in cotton leaf tissues (Table 1). For example, heat-stressed Sicot 71BRF and 5B plants had 25 and 20% (averaged across heat shock and ramping heat) higher *RCI* at 40 and 55°C, respectively, compared with control plants. AVG had no significant effect on leaf *RCI* of any cotton genotype under any temperature, except *RCI* of heat-shock plants at 40°C, where AVG treated leaves had significantly lower *RCI* than non-AVG treated leaves at the same temperature.

In the 2nd experiment, *RCI* of Sicot 71BRF and CIM 448 was significantly increased by 36 or 45°C treatments but the effect of 45°C was relatively >36°C (Table 2). ACC further increased the *RCI* of heat-stressed plants (Supplementary Table 5).

### *In Vitro* pollen development

Cotton plants exposed to heat shock or ramping heat for 1 week did not produce any viable pollen up to 2 weeks after the termination of stress. Therefore, pollen germination was tested only from the plants growing under control conditions. Similarly, no significant effect of prior treatment of plants with AVG or ACC was observed on subsequent pollen germination (data not shown).

**Figure 4:**
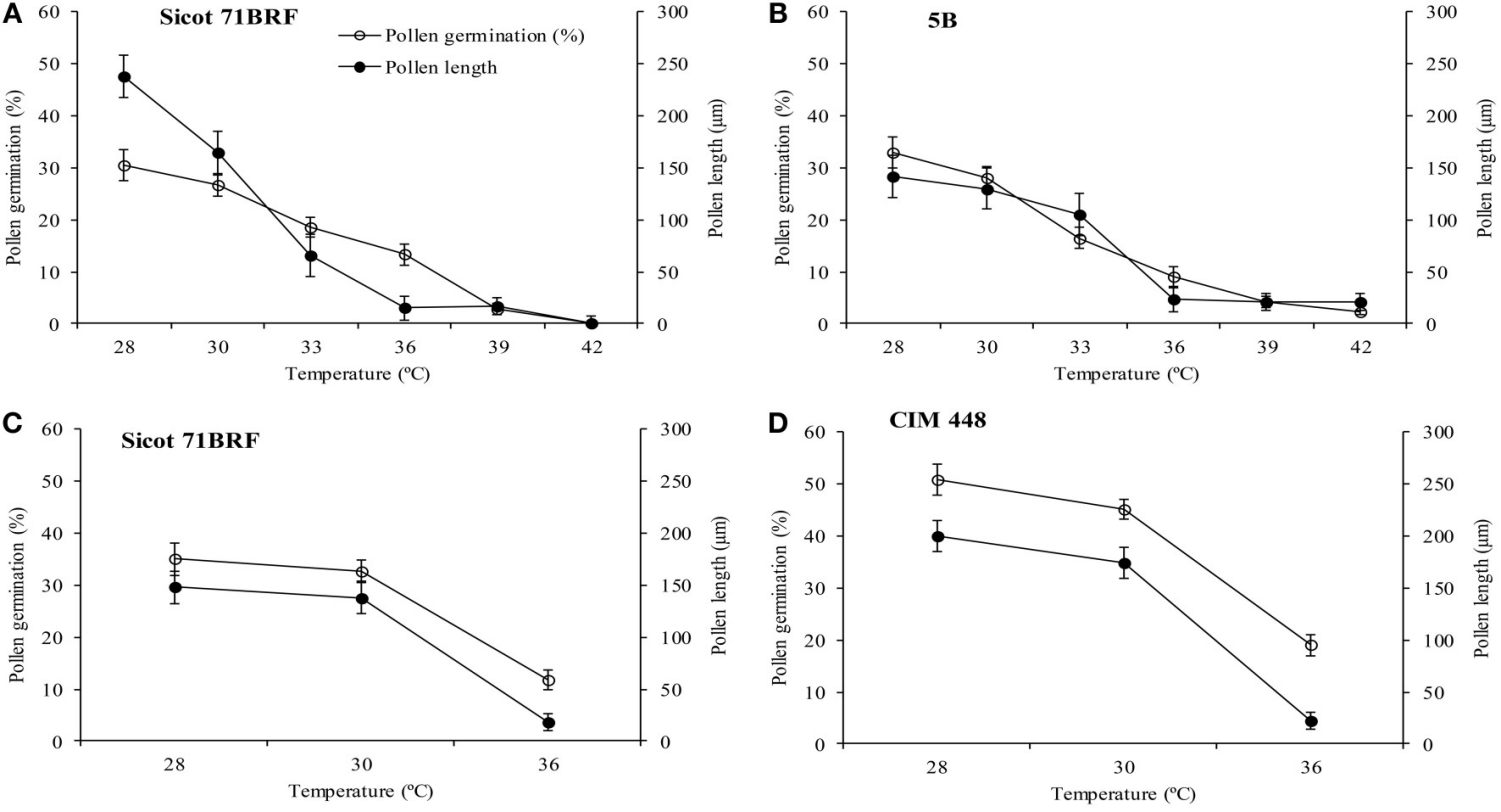
Pollen germination and pollen tube length of **(A)** Sicot 71BRF **(B)** 5B genotypes 24 h after *in vitro* incubation under varying temperatures in experiment 1. Pollen germination and pollen tube length of **(C)** Sicot 71BRF **(D)** CIM 448 genotypes 24 h after *in vitro* incubation under varying temperatures in experiment 2. Values are the mean of four independent replications, with the error bars representing SE and sample size = 4.

No significant change in pollen germination percentage of the two genotypes (Sicot 71BRF and 5B) was observed by increasing incubation temperature from 28 to 30°C, however, further increase in incubation temperature significantly reduced the pollen germination rate, and no pollen germination was observed at ≥39°C (Figures 4A,B). Pollen tube growth was also inhibited by increasing temperature over the same range.

In the 2nd experiment, pollen germination was tested only at three temperatures (28, 30, and 36°C). Pollen germination and pollen tube length of Sicot 71BRF exhibited a similar trend to that of the first experiment, showing no significant change by increasing incubation temperature from 28 to 30°C and a three-fold reduction at 36°C. CIM 448 plants had significantly greater pollen germination (%) than Sicot 71BRF under different temperatures; e.g., germination (%) of CIM 448 pollen was almost twice that of Sicot 71BRF at 36°C, although no significant difference in pollen tube length of both cotton genotypes was observed at this temperature (Figures 4C,D).

### Ethylene production

**Figure 5:**
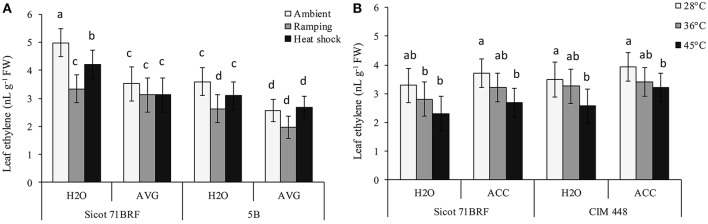
**(A)** Effect of aminoethoxyvinylglycine (AVG) on ethylene release from leaf tissues of cotton genotypes Sicot 71BRF and 5B exposed to heat shock (45°C) and ramping heat (gradual rise in temperature to 45°C) for 7 days, data were collected at the termination of heat treatment. **(B)** Effect of 1-aminocyclopropane-1-carboxylic acid (ACC) on ethylene release from leaf tissues of cotton genotypes Sicot 71BRF and CIM 448 exposed to heat shock (45 and 36°C) for 7 days, data were collected at the termination of heat treatment. Values are the mean of four independent replications, with the error bars representing SE and sample size = 4. Means sharing same letters within each figure are not significantly different at α = 0.05.

Elevated temperature (45°C) significantly reduced ethylene release from leaf tissues of cotton cultivars Sicot 71BRF and CIM 448, although its effect on 5B was non-significant (Figures 5A,B). Similarly, ramping high temperature significantly reduced ethylene release from leaf tissues of Sicot 71BRF and 5B genotypes and this reduction was relatively greater than that of heat-shock treatment. No significant change in ethylene release was observed in any cotton cultivar in response to 36°C. AVG also significantly reduced ethylene release from leaf tissues of cotton cultivars under all treatment conditions. Although, there was a significant increase in ethylene production from ACC-treated cotton leaves when measured 24 h after ACC application (data not shown), no significant change in ethylene production was observed at the termination of heat treatment (7 days after ACC application; Figure 5B).

### Relationship between fruit production and leaf physiology

**Figure 6:**
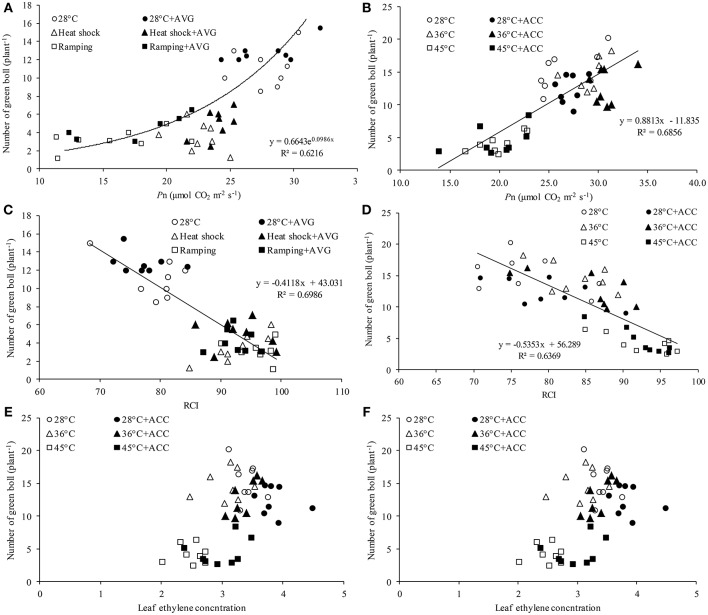
Relationship between green bolls and various physiological attributes of cotton genotypes under varying temperatures and chemical treatments. Relationship between green bolls and leaf photosynthesis (*P*_n_) of **(A)** genotypes Sicot 71BRF and 5B treated with 0 or 0.8 mM aminoethoxyvinylglycine (AVG) and **(B)** genotypes Sicot 71BRF and CIM 448 treated with 0 or 20 μM 1-aminocyclopropane-1-carboxylic acid (ACC). Relationship between green bolls and relative cell injury (RCI) of **(C)** genotypes Sicot 71BRF and 5B treated with 0 or 0.8 mM AVG and **(D)** genotypes Sicot 71BRF and CIM 448 treated with 0 or 20 μM ACC. Relationship between green bolls and ethylene concentration in the leaf tissues of **(E)** genotypes Sicot 71BRF and 5B treated with 0 or 0.8 mM AVG and **(F)** genotypes Sicot 71BRF and CIM 448 treated with 0 or 20 μM ACC.

The mechanism of heat-induced fruit loss was explored by studying the relationships between number of green bolls and the major yield-affecting variables e.g., leaf *P*_n_, ethylene and *RCI*. Irrespective of treatment conditions, number of green bolls in the studied genotypes showed a strong positive relationship with photosynthesis (Figures 6A,B) and a negative relationship with *RCI* (Figures 6C,D). In contrast, no significant correlation was observed between fruit number and leaf ethylene release in this study (Figures 6E,F).

**Figure 7:**
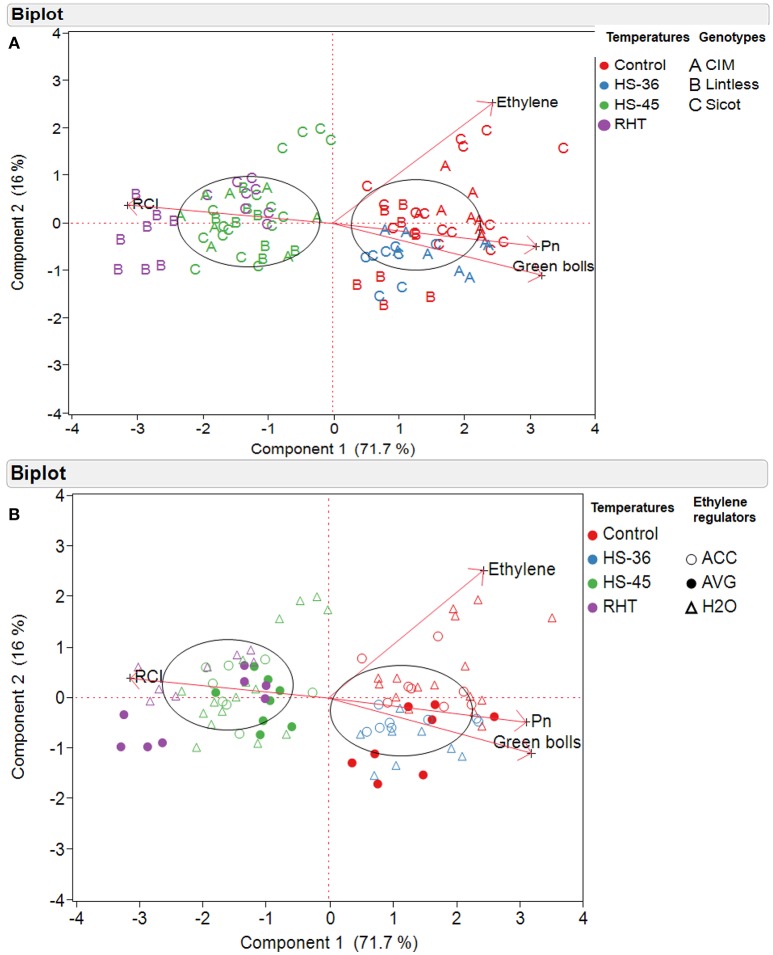
Principal component analysis (PCA) of cotton plants subjected to different temperatures, based on **(A)** cotton genotypes and **(B)** ethylene regulators. The temperature treatments were control (28), heat shock to 36°C (HS-36), heat shock to 45°C (HS-45) and ramping high temperature to 45°C (RHT). Ethylene regulators, including 1-aminocyclopropane-1-carboxylic acid (ACC), aminoethoxyvinylglycine (AVG) and water (H_2_O) were sprayed 1 day prior to heat treatment. A biplot shows two-dimensional loadings of various variables e.g., Ethylene (concentration youngest fully expanded leaves), *P*n (rate of photosynthesis in youngest fully expanded leaves), number of green bolls (per plant), and *RCI* (%, relative cell injury).

Relationships among various components was further explored using multivariate analysis. PCA was conducted based on cotton genotypes (Figure 7A) and ethylene regulator application (Figure 7B). The loading matrix of PCA also indicated a strong positive correlation between *P*_n_ and green bolls, which were negatively correlated with leaf *RCI* (Figures 7A,B). Variation caused by different treatments and genotypes were mainly explained by first principal components (71.7%, PC1) followed by second principal components (16%, PC2; Figures 7A,B). The eigenvectors for PC1 and PC2 were

$$\begin{array}{l} \text{PC1 }=\;0.536\text{X}1,\;0.522\text{X}2,-0.523\text{X}3,\;0.409\text{X}4\text{ and}\\ \text{PC2 }=\;-0.386\text{X}1,\;-0.169\text{X}2,\;0.138\text{X}3,\;0.896\text{X}4 \end{array}$$

where X1 is number of green bolls; X2 is leaf *P*_n_; X3 is leaf *RCI* and X4 is leaf ethylene production.

Significantly higher values of eigenvectors for leaf *P*_n_ and *RCI* indicated that PC1 is an index of greater number of fruits in cotton genotypes with high *P*_n_ and lower *RCI*, and it mainly separated the plants because of temperature treatments (Figures 7A,B). A distinctive clustering pattern of the variables was observed in PC1 e.g., the genotypes under control 28 and 36°C were clustered mainly on the right hand side of the biplot, showing higher number of green bolls and photosynthesis. On the other hand, the plants exposed to 45°C either through heat shock or ramping high temperature were clustered on the left-hand side, indicating higher level of *RCI*. PC2 was an index of the subtle differences in leaf ethylene and number of green bolls and thus could explain the separation of variables in terms of ethylene production.

## Discussion

Significant losses in cotton lint yield has been reported under high temperature in numerous glasshouse and field studies (Ehlig and Lemert, 1973; Kakani et al., 2005; Zhao et al., 2005). Reddy et al. (1991b) suggest that a rise in maximum day temperature above 30°C will induce abscission of squares and flowers, while cotton plants shed most of their fruits when temperatures rise above 40°C. The first objective of the experiments reported here was to identify the pattern of fruit loss in selected cotton genotypes in response to sudden and graduated exposure to high temperatures. Surprisingly, sudden exposure to high temperatures had less impact on fruit production than heat imposed after ramping, even though the duration of heating in ramping treatments was 11 d compared with 7 d in heat-shocked plants. In spite of this, the period that plants were held at 45°C was the same in both treatments and there was no evidence of acclimation when plants were raised to this temperature incrementally.

Plants were also surprisingly resilient after the heat-shock treatment. Plants exposed to 45°C supported significantly fewer squares at −1 DHT but they produced significantly more squares than non-stressed plants by 15 DHT since most of the squares in non-stressed plants had turned into green bolls at this stage. However, these young squares may not contribute to final lint yield due to delayed maturity, especially in mechanized cotton production systems, where cotton is harvested as a determinate crop (Da Costa and Cothren, 2011; Da Costa et al., 2011). On the other hand, plants in the ramped heat treatment had no more squares than control plants at 15 DHT, indicating that this long-term heat treatment irreversibly damaged reproductive development. This assertion was also supported by significantly greater reduction in *P*_n_ after ramping heat than after heat shock. Despite continuing square production, heat-shocked (45°C) plants were unable to produce a viable reproductive flower up to 15 DHT, indicating that high temperature specifically impaired the early phases of pollen development, leading to production of abnormal (sterile) pollen. This was supported by *in vitro* pollen germination assays, where heat-stressed plants did not produce any viable pollen at 15 DHT. Similar data have been proposed by Fisher (1975) who found that high temperature induced pollen sterility and subsequent fruit abscission in cotton during the late reproductive phase. Song et al. (2015) indicated that sporogenous cell to tetrad formation phases (16–24 days prior to anthesis) are the most sensitive stages of pollen development to high temperature. Thus, yield losses in heat-stressed cotton can be minimized by protecting early phases of pollen development. HSP101 protein plays a key role in thermotolerance acquisition in plants, however, the gene coding for this protein is not normally expressed in pollen tissues. Burke and Chen (2015) has achieved a degree of success in increasing thermotolerance in cotton and tobacco pollen through introduction of a gene (*AtHSP101*) encoding a heat shock protein. The resultant transgenic plants also exhibited better growth and yield stability compared with wild type plants at high temperature.

Reduced photosynthesis in response to high temperature (heat shock and ramping), as observed in this study, has been documented (Lu et al., 1997; Hejnák et al., 2015). However, in contrast to Perry et al. (1983) who suggested 33°C as a threshold temperature for diminished photosynthesis in cotton, leaf *P*_n_ was unchanged up to 36°C in Sicot 71BRF and CIM 448, with PSII yield unaffected and *g*_s_ significantly increased. This reflects the development of modern and more heat-tolerant cotton cultivars. Zhao et al. (2005) also observed undiminished *P*_n_ up to 36°C. Increasing *g*_s_ with temperatures up to 45°C show that stomatal resistance did not constrain *P*_n_ even at high temperatures, indicating that lower *P*_n_ was caused by other factors such as, temperature-induced injury to electron transport (Wise et al., 2004), inhibited activation of rubisco (Scafaro et al., 2016) or lower mesophyll conductance (Scafaro et al., 2011). However, negative effects of heat on *P*_n_ in 5B was seen after plants were ramped to 45°C, at least in part because of reduced stomatal closure. The PSII complex is very sensitive to abiotic stresses and elevated temperature can damage components of PSII complex of the thylakoid membranes by increasing fluidity of thylakoid membranes (Schrader et al., 2004). The current study in cotton also showed major decreases in PSII yield accelerated abscission of fruits. This finding was also supported by increased *RCI* in cotton leaves under elevated temperature (45°C) in the present study. Thus heat-induced impaired electron flow and energy transfer pathway is a likely reason for photosynthetic inhibition in cotton leaves (Cottee et al., 2014).

Heat-induced damage to cotton growth has been linked with peroxidation of lipid membranes (Bibi et al., 2008)—a key site for sensing high and low temperature conditions in plants (Örvar et al., 2000). As a result, cell membrane thermostability has often been used as an indicator of heat stress tolerance in cotton (Cottee et al., 2007). A strong negative correlation between green bolls and *RCI* in the present study supports the case for heat-induced cellular damage across all genotypes. For example, less fruit loss in Sicot 71BRF than 5B under ramping heat could be associated with more resilient membranes in the commercial genotype and better photosynthetic performance. Nonetheless, at 36°C fruits of Sicot 71BRF began to abort without any significant change in photosynthetic performance, indicating that photoassimilate partitioning or more specific effects in the reproductive machinery are also important in fruit set. In particular, genotypic variation in reproductive potential in response to higher temperature was closely associated with pollen viability, as suggested by Kakani et al. (2005), as seen in relatively better pollen germination in the more heat-tolerant genotype (CIM 448) under 36°C.

The second objective of these experiments was to establish a link between ethylene and fruit abscission in heat-stressed cotton. Despite numerous reports on the effect of anti-ethylene agents (e.g., 1-MCP) on heat-stressed cotton (Oosterhuis et al. (2009), limited work has been done to practically measure ethylene concentrations from cotton tissues (Kawakami et al., 2013). In contrast to wheat (Hays et al., 2007) and soybean (Djanaguiraman et al., 2011), where high temperature induces ethylene production, significantly lower ethylene concentration in heat-stressed cotton tissues was recorded in this study. Similarly, Kawakami et al. (2013) observed that heat stress can significantly reduce ethylene production from reproductive tissues of cotton (38/20°C) 2 days after anthesis and proposed that fruit development could be increased by regulating ethylene metabolism. However, significant loss of fruits in heat-stressed plants despite lower ethylene concentration in the present study suggested a limited role of ethylene in regulating heat-induced damage to cotton fruits. This was supported by the observation that there was no significant effect of AVG or ACC (ethylene regulators) on fruit production in cotton under high temperatures.

PCA indicated that clustering of variables on PC1 (72% of total variations) was mainly controlled by temperature rather than chemical application. A negative relationship (eigenvectors) between leaf ethylene and green bolls on PC2 (16% of total variations) indicates that ethylene might play a minor role reducing fruit number of cotton. However, this separation on PC1 was mainly observed in Sicot 71BRF under 28°C (Figures 7A,B), and can be explained by the positive effects of AVG on fruit production rather than ethylene-induced fruit loss under control conditions. AVG was effective in increasing fruit production in non-stressed cotton via improved carbon assimilation (Najeeb et al., 2015). Although positive effects of an ethylene action inhibitor (1-MCP) has been reported on the growth and yield of heat stressed cotton, no data are available on the effect of ethylene synthesis inhibitor (AVG) on heat-stressed cotton. For example, Kawakami et al. (2010) suggested that 1-MCP can increase lint yield of heat-stressed cotton by increasing boll weight but not fruit abscission, while Oosterhuis et al. (2009) observed significantly more fruits in heat-stressed cotton in response to 1-MCP application. Similarly, Chen et al. (2015) and Kawakami et al. (2013) observed positive effects of 1-MCP on lipid membrane and leaf chlorophyll of heat-stressed cotton but Scheiner et al. (2007) observed no significant effect of 1-MCP on these parameters. Thus, it is hard to conclude that inhibiting ethylene production or action in cotton tissues can protect cotton crops from heat-induced injury. Further, most of these 1-MCP application experiments were conducted under field conditions (where potentially multiple stresses including drought are present), which complicates the role of anti-ethylene agent in protecting cotton crops from high temperatures.

Based on the inhibitory role of ethylene at high temperatures, we further tested whether elevated ethylene may protect cotton from heat-induced injury. Earlier studies suggested positive effects of ethylene on heat-stressed plants e.g., *Arabidopsis* (Larkindale and Knight, 2002) and tomato (Firon et al., 2012). In the present study, ACC slightly increased *g*_s_ and *P*_n_ of heat-stressed plants, indicating that ACC might regulate stomatal behavior of cotton by modulating ABA synthesis (Ahmed et al., 2006). In addition, it increased green bolls of cotton at 15 DHT under 28 and 36°C. However, it is difficult to conclude that this increase was induced by ACC treatment, since no significant increase in leaf ethylene was detected at the end of heat treatment. Instead, accelerated fruit production in ACC treated plants at 15 DHT could be result of tendency of cotton plants to compensate ACC-induced early loss of flowers (Stewart et al., 2001). This was also supported by production of significantly more squares at −1 DHT (7 days after ACC application) but the effect became non-significant at 15 DHT.

## Conclusions

Elevated temperature (45°C) significantly impaired various physiological processes of cotton, leading to pollen sterility and flower abscission. Cotton plants increased leaf stomatal conductance but could not protect photosynthetic machinery from heat-induced (45°C) injury. High temperature and AVG reduced ethylene production, while ACC caused a transitory increase in ethylene production from cotton tissues but these treatments (ACC or AVG) could not affect abscission of fruits. Similarly, the ethylene-defective genotype 5B had an impaired tolerance to heat when compared with the commercial genotypes tested. Further heat-stressed (45°C) cotton plants (irrespective of ACC or AVG treatment) did not produce any viable pollen up to 2–3 weeks after stress, indicating that ethylene might not play any direct role in heat-induced yield damage to cotton. In contrast, high temperature induces fruit abscission either directly by impairing pollen development and/or indirectly by inhibiting photoassimilate supply to developing fruits.

## Author contributions

Concept development, experimental planning, revision of initial draft: DT, MB, and BA. Experimental execution, data collection, analysis, and initial writing: UN and MS.

### Conflict of interest statement

The authors declare that the research was conducted in the absence of any commercial or financial relationships that could be construed as a potential conflict of interest.
