# Supplementary material for: Endogenous Ethylene Concentration Is Not a Major Determinant of Fruit Abscission in Heat-Stressed Cotton (Gossypium hirsutum L.)
Source: Front Plant Sci. 2017 Sep 21;8:1615. doi: 10.3389/fpls.2017.01615 (PMC5613130; doi:10.3389/fpls.2017.01615)
Supplement: Supplementary file 1 [file Table1.DOCX]

**Table 1:** Treatment plan for Experiments 1 & 2.

| **Experiment 1** | | | | | | | | |
| --- | --- | --- | --- | --- | --- | --- | --- | --- |
| AVG | 0 mM | 0 mM | 0 mM | | 0.8 mM | 0.8 mM | 0.8 mM | |
| Temperature | 28^o^C  (optimum) | 45^o^C  (heat shock) | 45^o^C  (ramping heat) | | 28^o^C  (optimum) | 45^o^C  (heat shock) | 45^o^C  (ramping heat) | |
| **Experiment 2** | | | | | | | | |
| ACC | 0 µM | 0 µM | 0 µM | 20 µM | | 20 µM | | 20 µM |
| Temperature | 28^o^C  (optimum) | 36^o^C  (heat shock) | 45^o^C  (heat shock) | 28^o^C  (optimum) | | 36^o^C  (heat shock) | | 45^o^C  (heat shock) |

**Table 2**: Analysis of variance of the effect of treatments e.g. temperature, AVG (aminoethoxyvinylglycine) and ACC (1-aminocylopropane-1-carboxylic acid) on fruit numbers in cotton genotypes. *P* values are determined by multiple comparisons using Tukey HSD test, and *P* < α = 0.05 indicate statistical significance. Data were collected 1 and 15 days after termination of the heat treatment (DHT).

| **Experiment 1** | **Number of green bolls** | | **Number of squares** | | **Experiment 2** | **Number of green bolls** | | **Number of squares** | |
| --- | --- | --- | --- | --- | --- | --- | --- | --- | --- |
|  | 1 DHT | 15 DHT | 1 DHT | 15 DHT |  | 1 DHT | 15 DHT | 1 DHT | 15 DHT |
| Genotype | **0.003** | **0.0001** | **0.035** | 0.242 | Genotype | 0.107 | 0.068 | **0.002** | 0.364 |
| Temp | **<.0001** | **<.0001** | **0.028** | **<.0001** | Temp | **<.0001** | **<.0001** | **<.0001** | **0.001** |
| AVG | 0.135 | 0.086 | 0.606 | 0.958 | ACC | 0.851 | 0.514 | **0.047** | 0.178 |
| Genotype*Temp | 0.222 | **<.0001** | 0.412 | 0.158 | Genotype* Temp | 0.462 | 0.118 | 0.129 | 0.224 |
| Genotype*AVG | 0.697 | 0.448 | 0.185 | 0.958 | Genotype* ACC | 0.647 | 0.319 | 0.603 | 0.743 |
| Temp*AVG | 0.051 | **0.001** | 0.958 | 0.395 | Temp*ACC | 0.632 | 0.836 | 0.094 | **0.032** |
| Genotype*Temp*AVG | **0.035** | 0.098 | **0.023** | 0.081 | Genotype*ACC*Temp | 0.295 | 0.103 | 0.952 | 0.651 |

**Table 3**: Analysis of variance of the effect of treatments e.g. temperature, AVG (aminoethoxyvinylglycine) and ACC (1-aminocylopropane-1-carboxylic acid) on fruit retention in cotton genotypes. *P* values are determined by multiple comparisons using Tukey HSD test, and *P* < α = 0.05 indicate statistical significance. Data were collected 1 and 15 days after termination of the heat treatment (DHT).

| **Experiment 1** | **Fruit retention** | | **Experiment 2** | **Fruit retention** | |
| --- | --- | --- | --- | --- | --- |
|  | 1 DHT | 15 DHT |  | 1 DHT | 15 DHT |
| Genotype | 0.524 | 0.245 | Genotype | 0.271 | 0.328 |
| Temp | **<.0001** | **<.0001** | Temp | **<.0001** | **0.001** |
| AVG | 0.158 | 0.284 | ACC | **0.015** | 0.0.84 |
| Genotype*Temp | **0.022** | **0.015** | Genotype*Temp | 0.584 | 0.258 |
| Genotype*AVG | 0.128 | **0.044** | Genotype*ACC | 0.654 | 0.758 |
| Temp*AVG | 0.121 | 0.541 | Temp*ACC | 0.124 | 0.517 |
| Genotype*Temp*AVG | **0.001** | 0.147 | Genotype*ACC*Temp | 0.584 | 0.752 |

**Table 4**: Analysis of variance of the effect of temperature, AVG (aminoethoxyvinylglycine) and ACC (1-aminocylopropane-1-carboxylic acid) on leaf gas exchange and chlorophyll florescence in cotton genotypes. *P* values are determined by multiple comparisons using Tukey HSD test, and *P* < α = 0.05 indicate statistical significance. Data were collected at termination of the heat treatment (DHT).

| **Experiment 1** | ***P*_n_** | ***g*_s_** | **PSII** | **Experiment 2** | ***P*_n_** | ***g*_s_** | **PSII** |
| --- | --- | --- | --- | --- | --- | --- | --- |
| Genotype | **<.0001** | **<.0001** | 0.201 | Genotype | **<.0001** | **<.0001** | **<.0001** |
| Temp | **<.0001** | **<.0001** | **<.0001** | Temp | **<.0001** | **<.0001** | **<.0001** |
| AVG | 0.326 | 0.349 | 0.188 | ACC | **0.**152 | **0.460** | **0.275** |
| Genotype*Temp | **<.0001** | **<.0001** | **0.025** | Genotype*Temp | **0.039** | 0.670 | 0.124 |
| Genotype*AVG | 0.423 | **0.046** | 0.943 | Genotype*ACC | 0.156 | **0.251** | 0.656 |
| Temp*AVG | **0.001** | **0.001** | **0.024** | Temp* ACC | **0.026** | **<.0001** | 0.096 |
| Genotype*Temp*AVG | **0.001** | **0.005** | 0.193 | Genotype*ACC*Temp | 0.128 | 0.171 | 0.511 |

*P*_n_ = rate of photosynthesis; *g*_s_ = stomatal conductance; Tr = transpiration rate; PSII = efficiency of photosystem II

**Table 5:** Analysis of variance of the effect of temperature, AVG (aminoethoxyvinylglycine) and ACC (1-aminocylopropane-1-carboxylic acid) on leaf ethylene and RCI (relative cell injury) in cotton genotypes. *P* values are determined by multiple comparisons using Tukey HSD test, and *P* < α = 0.05 indicate statistical significance. Data were collected at termination of the heat treatment (DHT).

| **Experiment 1** | **RCI (%)** | **Ethylene** | **Experiment 2** | **RCI (%)** | **Ethylene** |
| --- | --- | --- | --- | --- | --- |
| Genotype | 0.777 | **0.041** | Genotype | 0.071 | 0.464 |
| Temp | **<.0001** | **0.042** | Temp | 0.670 | **0.031** |
| AVG | 0.321 | **0.001** | ACC | **<.0001** | 0.093 |
| Genotype*Temp | 0.475 | 0.054 | Genotype*ACC | 0.408 | 0.271 |
| Genotype*AVG | 0.057 | 0.126 | Genotype*Temp | **0.035** | 0.180 |
| Temp*AVG | 0.423 | 0.870 | ACC*Temp | 0.556 | 0.240 |
| Genotype*Temp*AVG | 0.273 | 0.819 | Genotype*ACC*Temp | 0.585 | 0.595 |
